# Omission of clinical target volume in radiotherapy for unresectable stage III non-small cell lung cancer: a propensity score matching analysis

**DOI:** 10.1080/07853890.2026.2650866

**Published:** 2026-03-30

**Authors:** Wenxin Ding, Jing Liang, Chaoying Chen, Jingsheng Zhao, Chaojiu Xu, Jinhua Zhang, Hao Zhou, Honghua Wu, Jian Long, Quan Zuo, Zhi Yang

**Affiliations:** Department of Oncology, People’s Hospital of Xiangxi Tujia and Miao Autonomous Prefecture, First Affiliated Hospital of Jishou University, Jishou, China

**Keywords:** Unresectable, stage III, non-small cell lung cancer, radiotherapy, CTV omission

## Abstract

**Objective:**

To investigate the efficacy and adverse reactions of radiotherapy with omitted clinical target volume (CTV) in unresectable stage III non-small cell lung cancer (NSCLC).

**Methods:**

A total of 423 NSCLC patients admitted to our hospital from June 2020 to April 2024 were enrolled. Among them, 112 patients met the inclusion criteria, including 58 patients in the experimental group and 54 patients in the control group. After 1:1 propensity score matching (PSM), 66 patients were included in the final analysis, with 33 patients in each group. All patients received radical radiotherapy and were divided into experimental and control groups based on whether CTV was omitted. The efficacy and adverse reactions between the two groups were analyzed.

**Results:**

The objective response rates were 45.4% in the experimental group and 57.5% in the control group (*p* = 0.325), showing no significant difference. Median locoregional recurrence-free survival (LRFS) was 28.2 months versus 23.2 months (*p* = 0.830). Median progression-free survival (PFS) was 21.3 months versus 17.3 months (*p* = 0.973). Median distant metastasis-free survival (DMFS) was not reached in the experimental group versus 20.9 months in the control group (*p* = 0.545). Median overall survival (OS) was not reached in the experimental group versus 26.2 months in the control group (*p* = 0.496). None of these differences were statistically significant. Regarding adverse reactions, the incidence of grade ≥2 radiation pneumonitis was significantly lower in the experimental group (6.1%) than in the control group (24.2%, *p* = 0.039), while there was no significant difference in immune-related pneumonitis between the groups.

**Conclusion:**

In this cohort study of patients with unresectable stage III non-small cell lung carcinoma (NSCLC) undergoing radiation therapy, no statistically significant difference in survival was observed with omission of the CTV. However, omitting the CTV could reduce the incidence of grade ≥2 radiation pneumonitis, thereby alleviating the adverse reactions associated with this condition.

## 1. Introduction

About 30% of people with non-small cell lung cancer (NSCLC) are diagnosed at a locally advanced stage [1]. For unresectable locally advanced NSCLC, concurrent chemoradiotherapy (CCRT) has traditionally been the standard treatment, but the 5-year survival rate has long stagnated between 15% and 30% [2], indicating an urgent need for breakthroughs in treatment effectiveness. The PACIFIC study [3] and the GEMSTONE-301 study [4] changed this landscape.These studies demonstrated that immunotherapy consolidation after concurrent or sequential chemoradiotherapy significantly extended progression-free survival (PFS) and improved overall survival (OS) for unresectable locally advanced NSCLC, raising the 5-year survival rate to 42.9% [5]. These studies established immunotherapy following concurrent or sequential chemoradiotherapy as the new standard treatment for unresectable stage III NSCLC.

However, this treatment model still has significant limitations. In clinical practice, about 50% of stage III patients cannot receive radical chemoradiotherapy due to poor performance status (PS), excessively large radiation fields, or complications such as insufficient pulmonary reserve. Among those receiving chemoradiotherapy, about two-thirds undergo concurrent chemoradiotherapy, while one-third receive sequential chemoradiotherapy [6–8]. Furthermore, the cumulative toxicity from chemoradiotherapy substantially increases the risk of adverse effects during subsequent immunotherapy. The incidence of any grade of pneumonia was significantly higher in NSCLC patients receiving sequential immunotherapy following chest radiotherapy compared to those receiving chemoradiotherapy alone or immunotherapy alone [3,9]. Research by Zhang et al. found that the incidence of checkpoint inhibitor pneumonitis (CIP) in NSCLC is higher than in other tumor types, reaching 7%–13%; real-world incidence exceeds that observed in clinical studies [10]. Therefore, reducing the toxicity associated with chemoradiotherapy while maintaining its efficacy has become a significant challenge in optimizing the current NSCLC treatment model.

Research [11] found that the size of the planning target volume (PTV) is significantly related to the risk of radiation pneumonitis.The PTV is formed by expanding the clinical target volume (CTV). Specifically,the establishment of the CTV aims to treat gross tumor lesions and subclinical lesions as observed under microscopy. P Giraud et al. conducted a study on postoperative specimens from 70 patients with non-small cell lung cancer (NSCLC), measuring the distance between the gross tumor boundary and tumor invasion under microscopy. The results showed that to encompass 95% of subclinical lesions, the CTV for adenocarcinoma and squamous carcinoma needs to expand the gross tumor boundary by 8 mm and 6 mm, respectively [12]. To reduce the toxicity of radiotherapy, the possibility of omitting the clinical target volume (CTV) during treatment has been explored. Xia et al. [13] studied 13 patients with locally advanced NSCLC who underwent intensity-modulated radiotherapy (IMRT) plans that excluded the CTV, and the results showed that the 50 Gy isodose line covered at least 95% of the GTV plus the subclinical areas in all patients. These IMRT plans still delivered sufficient dose to the subclinical areas while reducing exposure to normal tissues. Omitting the CTV in NSCLC radiotherapy is feasible;however,further related research is still insufficient. This study aims to explore the efficacy and adverse reactions of omitting the CTV in radiotherapy for unresectable stage III NSCLC, providing solid evidence for treatment strategy selection.

## 2. Materials and methods

### 2.1. General information

**Figure 1.**
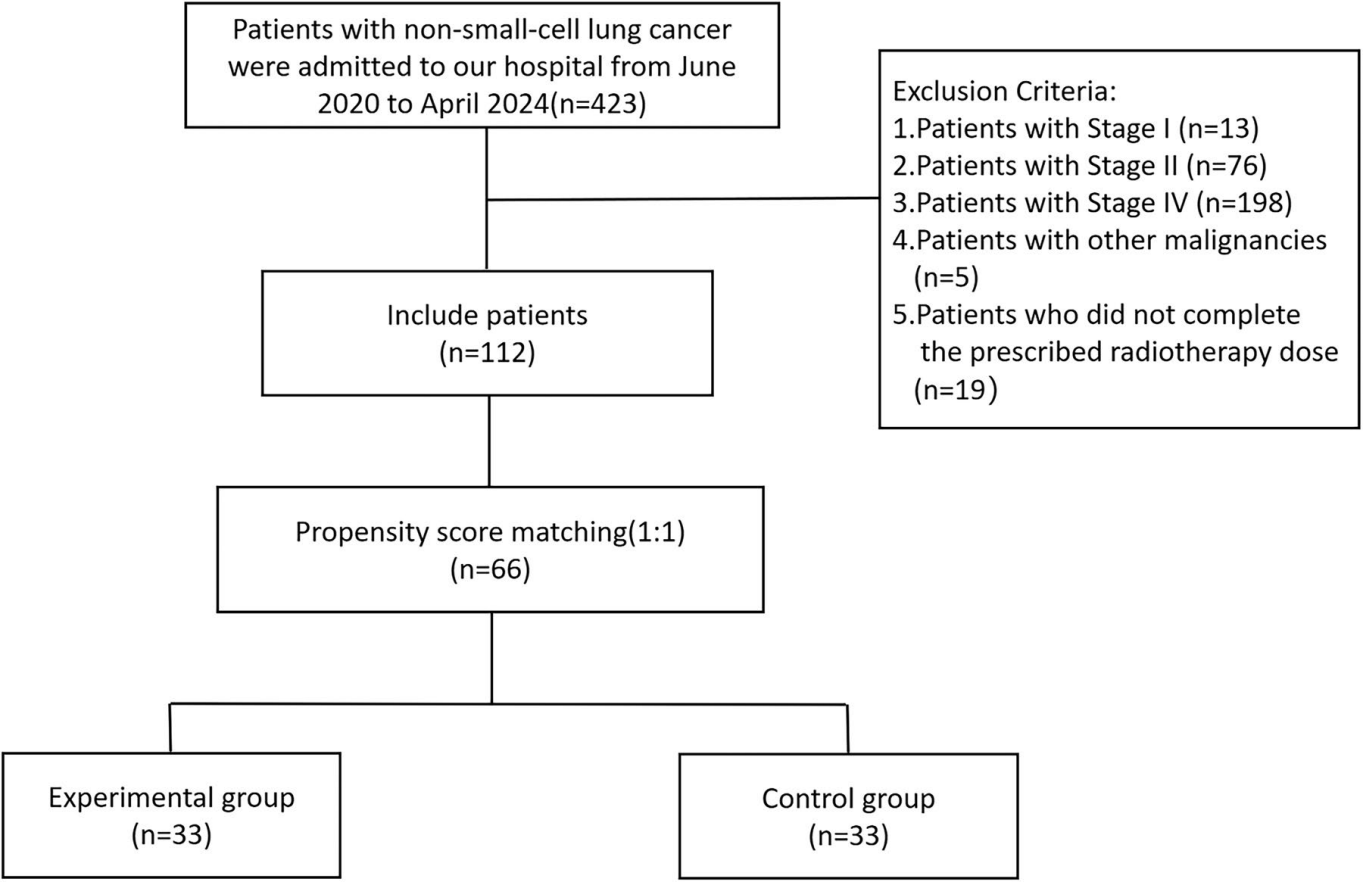
Flowchart of patient inclusion.

We gathered data on 423 cases of non-small cell lung cancer treated at our hospital from June 2020 to April 2024.Among these, 112 patients met the inclusion criteria, comprising 58 patients in the experimental group and 54 patients in the control group. After performing 1:1 propensity score matching (PSM) based on covariates including age, gender, history of chronic obstructive pulmonary disease (COPD), history of smoking, pathological type, T stage, N stage, clinical stage, history of induction therapy, history of concurrent therapy, and history of consolidation therapy, we included 66 patients (33 from each group) in the final analysis (Figure 1).

Inclusion criteria: 1) Confirmed non-small cell lung cancer through pathology; 2) Staged as unresectable stage III (AJCC 8th edition staging); 3) Concurrent chemoradiotherapy (at least 2 cycles of platinum-based chemotherapy during radiotherapy) or sequential chemoradiotherapy.

Exclusion criteria: 1) Patients with cancer stages other than stage III; 2) Patients with other malignancies; 3) Patients who did not complete the prescribed radiotherapy dose.

This study was conducted in accordance with the Declaration of Helsinki and was approved by the Ethics Committee of Xiangxi Autonomous Prefecture People’s Hospital (Approval No:EC-LCKY2025073). Due to the retrospective nature of the study, patient informed consent was waived.

### 2.2. Treatment methods

All patients received intensity-modulated radiation therapy (IMRT). Based on whether the radiation target area included the clinical target volume (CTV), patients were divided into the experimental group and the control group, with 33 cases in each group.

Induction treatment regimens included TP (paclitaxel + cisplatin/carboplatin) with or without PD-1 inhibitors, PC (pemetrexed + cisplatin/carboplatin). However, not all patients received induction therapy.

Both groups of patients received the same intensity-modulated radiation therapy (IMRT) technique, with target volume delineation and radiation dose delivered according to the ICRU83 report.In the experimental group, the gross tumor volume of the primary tumor (GTVp) plus the internal margin forms the internal target volume (ITV). The ITV was expanded by 5 mm to form the planning gross tumor volume (PGTVp, also referred to as PGTVp-g). Similarly, the gross tumor volume of the lymph node tumor (GTVnd) plus the internal margin formed the internal target volume (ITV). The ITV was expanded by 5 mm to form PGTVnd-g.In addition, the GTVp was expanded by 6 mm (for squamous cell carcinoma) or 8 mm (for adenocarcinoma) to construct the virtual clinical target volume of the primary tumor. The virtual clinical target volume of the lymph nodes (CTVnd) was formed by expanding GTVnd by 5 mm. When expanding the clinical target volumes of the primary tumor and lymph nodes, appropriate adjustments were applied to organs at risk such as the vertebral body, trachea, heart, and esophagus. The clinical target volume plus internal margin plus 5 mm formed the virtual planning target volume (PTVp-c and PTVnd-c). The aforementioned PTVp-c and PTVnd-c did not receive the prescribed doses.

In the control group, PGTVp-g and PGTVnd-g were delineated according to the same principles. The primary tumor CTV was formed by expanding the GTVp by 6 mm for squamous cell carcinoma and by 8 mm for adenocarcinoma, and the lymph node CTVnd was formed by expanding the GTVnd by 5 mm. Appropriate adjustments were also applied to organs at risk such as the vertebrae, trachea, heart, and esophagus when expanding the CTV. The CTV plus internal margin plus 5 mm formed PTVp-c and PTVnd-c, which received prescribed doses.

The doses for each target area with 95% dose coverage are as follows: the primary tumor PTV (PGTVp-g) is 60–66 Gy/30 fractions; the lymph node metastatic PTV (PGTVnd-g) is 60–66 Gy/30 fractions; the clinical target area for the primary tumor (PTVp-c) is 50–54 Gy/30 fractions; and the clinical target area for lymph nodes (PTVnd-c) is 50–54 Gy/30 fractions.

Concurrent chemotherapy regimens included TP, PC, EP (etoposide + cisplatin), or radiotherapy alone.

Consolidation therapy was conducted using PD-1 inhibitors or targeted drugs.

### 2.3. Efficacy and observation indicators

Short-term efficacy: A comprehensive re-examination was conducted 3 months after the end of concurrent chemoradiotherapy to assess short-term efficacy. The maximum diameter of the tumor was measured through imaging examinations, and changes in tumor diameter after treatment relative to baseline were evaluated according to the internationally accepted Response Evaluation Criteria in Solid Tumors (RECIST) version 1.1. Efficacy determination included complete response (CR), partial response (PR), stable disease (SD), and progressive disease (PD). The objective response rate (ORR) was defined as the proportion of patients achieving either CR or PR.

Adverse reactions: We recorded any cases of radiation pneumonia and immune pneumonia during and after radiotherapy, graded according to the international common adverse reaction standards (version 5.0).

Long-term efficacy: Follow-ups were conducted to monitor local recurrence, distant metastasis, and survival status. The primary observation indicator was locoregional relapse-free survival (LRFS), defined as the time from the start of treatment to the first occurrence of local recurrence or death from any cause, with local recurrence categorized into three types: within the PGTV-g range, between PGTV-g and PTV-c, and outside the PTV-c range. Secondary observation indicators included distant metastasis-free survival (DMFS), defined as the time from the start of treatment to the first occurrence of distant metastasis or death from any cause; progression-free survival (PFS), defined as the time from the start of treatment to the first occurrence of disease progression or death from any cause; and overall survival (OS), defined as the time from the start of treatment to death from any cause.

### 2.4. Follow-up observation

After the patient’s radical radiation therapy, the frequency of follow-up examinations in the first two years was once every three months; during the period of two to five years, follow-up examinations were conducted once every six months; after five years, the frequency of follow-up examinations was once a year. The follow-up items include enhanced chest CT, enhanced abdominal CT or abdominal color ultrasound, enhanced magnetic resonance imaging (MRI) of the brain, tumor markers, etc. When the patient exhibits obvious clinical symptoms, such as persistent cough, chest pain, weight loss, etc., relevant follow-up examinations should be conducted in a timely manner. The follow-up deadline is December 30, 2024. The patient’s follow-up time range is from 3 to 58 months, with a median follow-up time of 23.1 months.

### 2.5. Statistical analysis

SPSS 26.0 statistical software was used for 1:1 PSM matching with a caliper value of 0.02 and for statistical data analysis. The log-rank test was employed for univariate analysis. The Cox proportional hazards model was used for multivariate analysis to compare survival outcomes between different groups. Baseline characteristics and the incidence of adverse reactions were analyzed using Chi-square tests(with or without continuity correction) or Fisher’s exact test.If the measurement data are normally distributed and satisfy the test for homogeneity of variances, then, the t-test was used, and the data were represented as mean ± standard deviation. If the data wre not normally distributed, the Wilcoxon rank-sum test was used, and the data were represented as median (interquartile range). *p* < 0.05 was considered statistically significant.

## 3. Results

### 3.1. Comparison of baseline data between two groups

Prior to propensity score matching (PSM), there were significant differences between the experimental group and the control group in gender, smoking history, and N staging (*p* < 0.05). However, no significant differences between the two groups were observed in age, chronic obstructive pulmonary disease (COPD) history, pathological type, clinical staging, and T staging, nor in induction therapy, concurrent chemoradiotherapy, and consolidation therapy (*p* > 0.05).

After PSM, the experimental and control groups were comparable regarding age, gender, COPD history, smoking history, pathological type, clinical staging, T staging, N staging, induction therapy, concurrent chemoradiotherapy, and consolidation therapy (*p* > 0.05), as seen in Table 1.

**Table 1.** characteristics of the original and PSM cohorts.

	Original cohort			Propensity-score matched cohort		
Characteristics	Control group (*n* = 54) (%)	Experimental group (*n* = 58) (%)	P	Control group (*n* = 33) (%)	Experimental group (*n* = 33) (%)	P
Age (years)			0.386			0.792
<60	18 (33.3)	15 (25.8)		11 (33.3)	10 (30.3)	
≥60	36 (66.7)	43 (74.2)		22 (66.7)	23 (69.7)	
Gender			0.003			1.000
Male	51(94.4)	43 (74.1)		31 (93.9)	30 (90.9)	
Female	3 (5.6)	15 (25.9)		2 (6.1)	3 (9.1)	
COPD history			0.935			1.000
No	51 (94.4)	56 (96.6)		31 (93.9)	32 (97.0)	
Yes	3 (5.6)	2 (3.4)		2 (6.1)	1 (3.0)	
Smoking history			0.014			0.786
No	10 (18.5)	23 (39.7)		9 (27.3)	10 (30.3)	
Yes	44 (81.5)	35 (60.3)		24 (72.7)	23 (69.7)	
Pathologic type			0.734			1.000
Squamous carcinoma	44 (81.5)	44 (75.9)		27 (81.8)	27 (81.8)	
Adenocarcinoma	9 (16.7)	12 (20.7)		5 (15.2)	6 (18.2)	
Lymphoepithelioid carcinoma	1 (1.8)	2 (3.4)		1 (3.0)	0 (0.0)	
Clinical stage			0.187			1.000
IIIA	18 (33.3)	15 (25.8)		8 (24.2)	8 (24.2)	
IIIB	30 (55.6)	29 (50.0)		19 (57.6)	19 (57.6)	
IIIC	6 (11.1)	14 (24.1)		6 (18.2)	6 (18.2)	
T stage			0.382			0.297
T1	0 (0.0)	3 (5.2)		0 (0.0)	2 (6.1)	
T2	12 (22.2)	14 (24.1)		10 (30.3)	7 (21.2)	
T3	11 (20.4)	10 (17.2)		8 (24.2)	5 (15.2)	
T4	31 (57.4)	31 (53.4)		15 (45.5)	19 (57.5)	
N stage			0.028			0.330
N0	6 (11.1)	1 (1.7)		0 (0.0)	1 (3.0)	
N1	3 (5.6)	4 (6.9)		0 (0.0)	2 (6.1)	
N2	35 (64.8)	30 (51.7)		23 (69.7)	19 (57.6)	
N3	10 (18.5)	23 (39.7)		10 (30.3)	11 (33.3)	
Induction treatment			0.571			0.150
No	20 (37.0)	21 (36.2)		14 (42.4)	10 (30.3)	
TP+PD1 inhibitors	16 (29.6)	17 (29.3)		10 (30.3)	12 (36.4)	
TP	13 (24.1)	18 (31.0)		6 (18.2)	11 (33.3)	
PC	5 (9.3)	2 (3.5)		3 (9.1)	0 (0.0)	
Concurrent treatment			0.685			0.717
No	16 (29.6)	20 (34.5)		11 (33.3)	10 (30.3)	
TP	25 (46.3)	29 (50.0)		15 (45.5)	19 (57.6)	
EP	11 (20.4)	7 (12.1)		5 (15.2)	3 (9.1)	
PC	2 (3.7)	2 (3.4)		2 (6.1)	1 (3.0)	
Consolidation treatment			0.936			0.773
No	27 (50.0)	31 (53.4)		19 (57.5)	18 (54.6)	
PD1 inhibitors	24 (44.5)	24 (41.4)		12 (36.4)	14 (42.4)	
Targeted therapy	3 (5.5)	3 (5.2)		2 (6.1)	1 (3.0)	

### 3.2. Comparison of recent treatment efficacy between two groups

In a study involving 66 patients with locally advanced non-small cell lung cancer, the experimental group (33 patients) had one case of complete response (CR) and 14 cases of partial response (PR). There were also 18 cases of stable disease (SD) and no cases of progressive disease (PD). The objective response rate, calculated as the sum of CR and PR cases divided by the total number of patients, was 45.4%. The control group (33 patients) had one case of CR, 18 cases of PR, 14 cases of SD, and no cases of PD, resulting in an objective response rate of 57.5%. Statistical analysis found no significant difference in the objective response rates between the two groups (*p* = 0.325). You can find the specific data in Table 2.

**Table 2.** Comparison of recent efficacy between the two groups.

Group	Total Cases	CR	PR	SD	ORR
		Cases (%)	Cases (%)	Cases (%)	Cases (%)
Experimental group	33	1 (3.0)	14 (42.4)	18 (54.5)	15 (45.4)
Control group	33	1 (3.0)	18 (54.5)	14 (42.4)	19 (57.5)
P value					0.325

### 3.3. Comparison of long-term effectiveness between two groups

**Figure 2.**
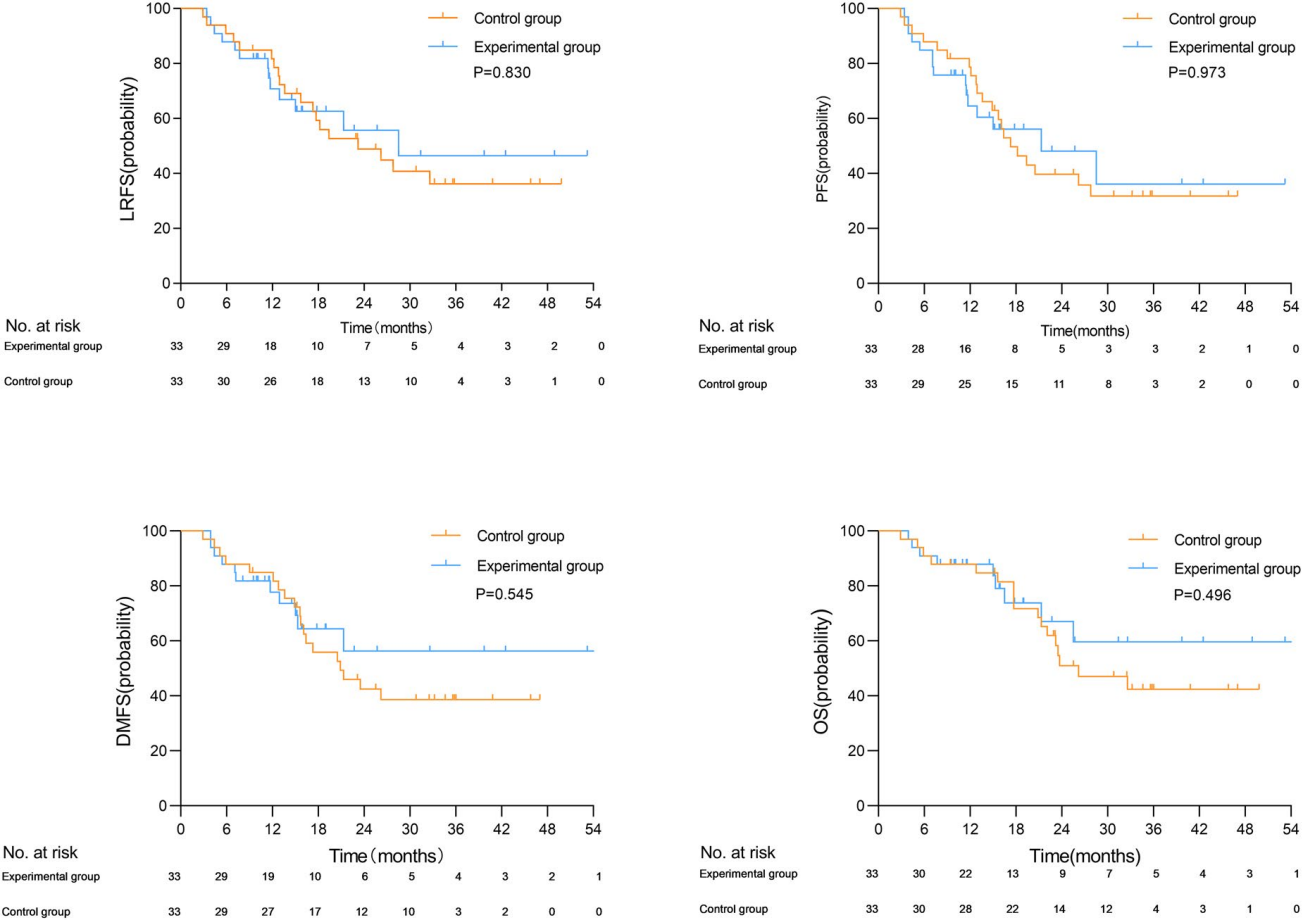
The Kaplan-Meier curves of local recurrence-free survival(LRFS), progression-free survival(PFS), distant metastasis-free survival(DMFS), and overall survival(OS) after PSM.

**Figure 3.**
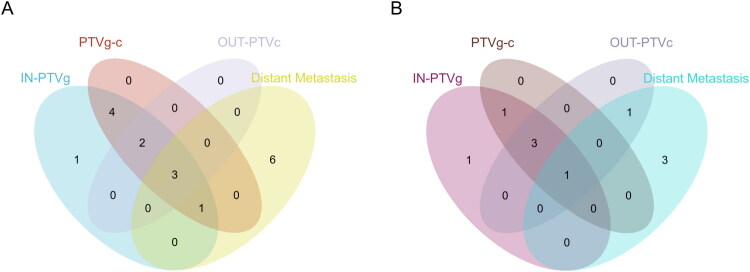
The patterns of first treatment failure in the control and experimental groups are shown in A and B.IN-PTVg is the recurrence within the range of PTVg.PTVg-c is the recurrence within the range of PTVg-c.OUT-PTVc is the local recurrence outside the PTVC range.

The median LRFS for the matched experimental group and control group was 28.2 and 23.2 months [95% CI, 10.5–35.9], respectively. Due to the small number of remaining risk patients at the median LRFS time point, it was not possible to calculate a reliable confidence interval for the experimental group (*p* = 0.830). The median PFS for the experimental group and control group were 21.3 months [95% CI, 6.8–35.8] and 17.3 months [95% CI, 12.9–21.7], respectively (*p* = 0.973). The median DMFS was not reached in the experimental group, while the control group had a median DMFS of 20.9 months [95% CI, 12.8–29.0] (*p* = 0.545). Similarly, the median OS was not reached in the experimental group, whereas the control group had a median OS of 26.2 months [95% CI, 15.5–36.9] (*p* = 0.496). These results are illustrated in Figure 2. Further analysis revealed 10 cases of disease progression in the experimental group, 7 cases of local recurrence (including: 1 case within the PTVg range, 1 case within the PTVg and PTVg-c ranges, 4 cases within both the PTVg and PTVg-c ranges and outside the PTVc range, and 1 case outside the PTVc range), 5 cases of distant metastasis, and 9 cases of death. In the control group, we observed 17 cases of disease progression, 11 cases of local recurrence (including: 1 case within the PTVg range, 5 cases within the PTVg and PTVg-c ranges, and 5 cases of recurrence that occurred both within the PTVg and PTVg-c ranges and outside the PTVc range), 10 cases of distant metastasis, and 17 cases of death. Refer to Figure 3.

### 3.4. Comparison of radiotherapy dosimetry between two groups

Comparison of the planning target volume (PTV), volume of bilateral lung receiving 5 Gy (V5), 20 Gy (V20), 30 Gy (V30), and mean lung dose (MLD) showed that the experimental group had significantly lower bilateral lung V5 (*p* = 0.024), MLD (*p* = 0.048), and PTV (*p* = 0.002) than the control group, while there were no significant differences in V20 and V30. Detailed results are presented in Table 3.

**Table 3.** Comparison of radiotherapy dosimetry between two groups.

Observation indicators	Experimental group	Control group	P
PTV(cc)	205 (150.2, 254.6)	295.4 (222.6, 387.2)	0.002
V5(%)	45.539 ± 10.067	50.494 ± 7.1333	0.024
V20(%)	22.3 (19.8, 26.1)	24.6 (21, 28)	0.178
V30(%)	15.4 (13.5, 18.1)	15.8 (13.74, 18.3)	0.513
MLD(Gy)	12.3 (10.4, 13.3)	12.8 (11.62, 14.7)	0.048

### 3.5. Comparison of adverse reactions between two groups

The main adverse reactions observed were radiation pneumonitis and immune pneumonitis. The results showed that no patients in either group experienced adverse reactions with grade 4 or higher. The experimental group had 13 cases of grade 1 radiation pneumonitis and 2 cases of grade 2 radiation pneumonitis. The control group had 10 cases of grade 1 radiation pneumonitis, 7 cases of grade 2 radiation pneumonitis, and 1 case of grade 3 radiation pneumonitis. In the experimental group, the incidence of grade 2 or higher radiation pneumonitis was 6.1%, which is significantly lower than the 24.2% in the control group (*p* = 0.039). The experimental group had 1 case of grade 2 immune pneumonitis, while the control group had 1 case of grade 1 immune pneumonitis and 1 case of grade 2 immune pneumonitis, showing no significant difference in immune pneumonitis rates between the two groups (*p* > 0.05). See Table 4 for more details.

**Table 4.** Comparison of adverse reactions between the two groups.

Adverse reactions	Control group (*n* = 33)			Experimental group (*n* = 33)		P
	Grade 1 (%)	Grade 2 (%)	Grade 3 (%)	Grade 1 (%)	Grade 2 (%)	
Radiopneumonitis	10 (30.3)	7 (21.2)	1 (3.0)	13 (39.4)	2 (6.1)	0.039*
Immune pneumonitis	1 (3.0)	1 (3.0)	0 (0.0)	0 (0.0)	1 (3.0)	1.000**

Note: * is the P value for the comparison of grade ≥2 radiation pneumonia between the two groups, ** is the P value for the comparison of grade ≥1 immune pneumonitis between the two groups.

## 4. Discussion

No statistically significant difference was observed in median LRFS and PFS between the CTV-omitted group and the CTV-included group in this study.However, there is a lower incidence of radiation pneumonitis of grade 2 or higher,in the omission group. This suggests that omitting CTV in radiotherapy for inoperable stage III non-small cell lung cancer does not significantly affect patients’ local recurrence-free survival or progression-free survival;moreover, it can help reduce side effects.

Compared with NSCLC that can be converted to surgical resection after neoadjuvant chemotherapy [14], the patients in this study are those with NSCLC that cannot be converted to surgery, and have a worse prognosis.Chemoradiotherapy is the standard treatment for inoperable stage III non-small cell lung cancer, and due to the toxicity of chemoradiotherapy, about 50% of patients cannot undergo radical chemoradiotherapy in clinical practice [6]. Enhancing efficacy while reducing toxicity is a key challenge and focus in the research of inoperable stage III non-small cell lung cancer. The PACIFIC study [3] and the GEMSTONE-301 study [4] established a new approach for consolidative treatment with PD-L1 inhibitors after combined or sequential chemoradiotherapy in patients with unresectable stage III NSCLC. The LUN 14–179 study [15] showed that consolidative treatment with pembrolizumab after synchronous chemoradiotherapy also demonstrated good results. For inoperable stage III EGFR-mutant NSCLC, the LAURA study showed that consolidative treatment with osimertinib significantly prolonged progression-free survival and improved efficacy [16]. However, in the context of enhanced efficacy from chemoradiotherapy combined with immunotherapy or targeted therapy, side effects may also increase [17–18].

This study did not find that omitting CTV in radical radiotherapy shortens LRFS or PFS. Radiotherapy delivering radical doses (≥60Gy) to the lung cancer GTV also delivers a certain dose to surrounding subclinical lesions. For epithelial tumors, delivering a radiotherapy dose of 45-50Gy to subclinical lesions can achieve a high local control rate [19–20]. Xia et al. [13] conducted a dosimetric study on 13 patients with locally advanced NSCLC receiving intensity-modulated radiotherapy with CTV omission.They found that all patients had at least 95% of the GTV and CTV area covered by the 50 Gy dose line.Moreover, the IMRT plan without CTV delineation could provide sufficient radiotherapy dose coverage for subclinical lesions, providing dosimetric evidence supporting CTV omission in radiotherapy for locally advanced NSCLC.In addition, a study by Yuan et al. [21] on involved-field irradiation versus elective nodal irradiation for inoperable stage III NSCLC demonstrated that involved-field radiotherapy improves the prognosis of stage III NSCLC, and results in a lower incidence of adverse reactions. This study further reduces the irradiation volume compared to involved-field irradiation, preliminarily demonstrates that smaller-volume radiotherapy for lung cancer reduces treatment-related toxicity while maintaining local control. Kuncman et al. [22] also explored radiotherapy treatment models based on standard chemotherapy and immunotherapy for extensive small cell lung cancer. This study supports the broader modern thoracic radiotherapy strategy of limiting unnecessary target volumes in the era of intensified systemic therapy and immunotherapy. Although the study subjects were patients with extensive small cell lung cancer, it provides a conceptual theoretical background for this research. When applying the strategy of omitting the clinical target volume (CTV) in radiotherapy, it is necessary to weigh the potential risk of marginal recurrence associated with this approach.Zou et al. [23] performed a non-parallel controlled retrospective study on LA-NSCLC patients receiving synchronous chemoradiotherapy, finding that omitting CTV did not increase the risk of local recurrence, similar to the results of this study. Additionally, Liu et al. [24] also conducted a retrospective study on radiotherapy omitting CTV in locally advanced lung cancer.They reported that omission of CTV delineation did not adversely affect progression-free survival in LA-NSCLC patients, similar to the PFS results of this study.However,their study included very few patients who received immunotherapy consolidation after chemoradiotherapy.

Moreover, this study shows that after 1.5 years of follow-up, the overall survival in the CTV omission group tends to be higher than that of the CTV inclusion group.This difference may be related to some patients in this study receiving consolidation immunotherapy. Radiation-induced lymphopenia (RIL) is an immunosuppressive factor that can upset the delicate balance between anti-tumor immunity and immunosuppression [25]. Kuncman et al. [26] found that low-dose lung exposure (especially lung V5 and V10) and early dynamic changes in lymphocytes can predict radiation-induced lymphopenia during thoracic radiotherapy. Moreover, the lower the low-dose exposure, the lower the incidence of radiation-induced lymphopenia. Additionally,Zou et al. [23] found that lymphopenia during radiotherapy was related to the volumes of GTV and PTV; the larger the volume, the more severe the lymphopenia. Radiation-related lymphopenia increases the risk of the risk of mortality from lung cancer [27] and reduces the efficacy of immunotherapy [28]. This study omitted CTV, and the radiotherapy involved a smaller PTV and lung V5 volume, thereby reducing the occurrence of lymphopenia. This may have improved the overall survival in the CTV-omission group.

Omitting CTV reduces the volume of lung tissue exposed to radiation, which thereby reduce the incidence of radiation pneumonitis. This study shows a significant reduction in symptomatic radiation pneumonitis (grade ≥ 2), whereas the incidence of immune pneumonitis is comparable. Liu et al. [24] reported an even lower incidence of grade ≥3 radiation pneumonitis. Both radiotherapy and immunotherapy induce various cells to release multiple cytokines that play different roles in lung injury. Among these cytokines, IL-4, IL-6, IL-10, and IL-17 have been shown to be associated with both radiation-related pneumonitis and immune pneumonitis related to anti-PD-1/PD-L1 inhibitors.This suggests that the combination of radiotherapy and immunotherapy may increase the risk of both radiation pneumonitis and immune pneumonitis [29]. Geng et al. found that the combination of PD-1/PD-L1 inhibitors with radiotherapy in the treatment of non-small cell lung cancer could increase the incidence of grade 1–2 immune-related or radiation pneumonitis [30]. The above studies differ slightly from the results of this trial,which may be attributed to the relatively small sample size of this study,as it did not observe enough cases of grade ≥3 radiation pneumonitis and immune pneumonitis.

This study provides clinical evidence for a treatment plan that omits the CTV in radiotherapy. The study focuses on patients with unresectable locally advanced non-small cell lung cancer. However, this study has limitations. First, it is a single-center, small sample size retrospective study; the follow-up time is short, and the statistical power for comparing curative effects is insufficient, especially in assessing the risk of marginal local recurrence after the omission of the CTV. Additionally, adverse reactions—such as radiation-induced esophagitis and radiation-induced heart injury—that occurred during the follow-up period were not evaluated. Second, the general lack of PD-L1 status in this study is an important unmeasured confounding factor that may affect our precise interpretation of the efficacy of the immunotherapy subgroup. Third, patients who ‘did not complete the prescribed radiotherapy dose’ were excluded from this study. In clinical practice, incomplete administration of the prescribed dose is usually due to severe acute toxicity or rapid disease progression. Excluding these patients undoubtedly leads to an underestimation of the true incidence of toxicity in both groups and may introduce selection bias. This bias is particularly relevant for the control group, which may have a higher risk of toxicity, potentially underestimating the safety advantage of the experimental group.Although this study applied propensity score matching (PSM) to balance the baseline characteristics, the baseline included various therapeutic strategies. Therefore, heterogeneity in baseline characteristics may still exist, which warrants cautious interpretation.Future studies should enhance monitoring of adverse reactions and extend the follow-up time. Based on this, multi-center, randomized, large-sample, prospective studies should be conducted. Blind evaluation should also be used to confirm the research results.

## 5. Conclusion

In this cohort study of patients with unresectable stage III non-small cell lung carcinoma (NSCLC) undergoing radiation therapy, no statistically significant difference in survival was observed with omission of the CTV. However, omitting the CTV could reduce the incidence of grade ≥2 radiation pneumonitis, thereby alleviating the adverse reactions associated with this condition. In the context of chemoradiotherapy combined with immunotherapy, radiotherapy omitting the CTV for unresectable stage III non-small cell lung carcinoma may be a better treatment option.

## Data Availability

The datasets used and analysed during the current study are available from the corresponding author on reasonable request.
